# Performance Degradation Prediction Based on a Gaussian Mixture Model and Optimized Support Vector Regression for an Aviation Piston Pump

**DOI:** 10.3390/s20143854

**Published:** 2020-07-10

**Authors:** Chuanqi Lu, Shaoping Wang

**Affiliations:** 1 School of Engineering, Huazhong Agricultural University, Wuhan 430070, China; luchuanqi1987@buaa.edu.cn; 2 School of Automation Science and Electrical Engineering, Beihang University, Beijing 100191, China

**Keywords:** aviation piston pump, degradation index, support vector regression, prognostics

## Abstract

Performance degradation prediction plays a key role in realizing aviation pump health management and condition-based maintenance. Thus, this paper proposes a new approach that combines a Gaussian mixture model (GMM) and optimized support vector regression (SVR) to predict aviation pumps’ degradation processes based on the pump outlet pressure signals. Different from other feature extraction methods in which the information of intrinsic mode functions (IMFs) is not fully utilized, some useful IMF components are firstly chosen, and the corresponding multi-domain features are extracted from each selected component. Considering that it is not the case that all features are equally sensitive to degradation assessment, PCA is used to select more sensitive degradation features. Since the distribution of these extracted features is a stochastic process in feature space, meanwhile, self-information quantity can describe the uncertainty of system by measuring the average information quantity contained in the probability distribution, self-information quantity based on GMM is defined as degradation index (DI) to describe the degradation degree of the pump quantitatively. Finally, an SVR model is constructed to predict the degradation status of the pump. To achieve higher prediction accuracy, phase space reconstruction theory is first employed to determine the number of the inputs of the SVR model, then a new method combining particle swarm optimization (PSO) with grid search (GS) is developed to optimize the parameters of the SVR model. Finally, both the online data and historical data are utilized for the construction of the SVR model, respectively. The effectiveness of the proposed approach is validated by full life cycle data collected from an aviation pump test rig. The results demonstrate that the *DI* extracted from pump outlet pressure signals can effectively identify and track the current deterioration stage, and the established SVR model has better prediction ability when compared with previously published methods.

## 1. Introduction

The aviation pump, as one of the key components of the aircraft hydraulic system, provides high-pressure oil to the actuation system. Once an aviation pump fails, it will result in economic loss or even catastrophic consequences [1,2]. Hence, the safety and reliability of aviation pumps are crucial to the entire aircraft system [3]. Prognostics and health management (PHM) is a vital technology to improve the safety and reliability of the aviation pump. At present, some studies have been done on PHM of the aviation pump. Ma et al. [1] proposed a nonlinear unknown input observer to realize the fault diagnosis of the pump. Lu et al. [2] presented a multi-source information fusion method to improve the accuracy of the fault diagnosis of the pump. Du et al. [4] developed a layered clustering algorithm to diagnose the concurrent failures of the pump. From their papers, it can be found that most of the researches related to PHM of the pump focus on distinguishing different failure modes or fault sizes of the aviation pump. However, the goal of PHM is that not only could diagnose the faults but also could predict failures. As a matter of fact, an aviation pump usually experiences different degradation processes from normal to failure. This means that the key to failure prediction is to forecast the degradation processes of the pump accurately. So far, less study has been done on the research of performance degradation prediction of the aviation pump.

One of the main challenges of performance degradation prediction is how to create a suitable degradation index (*DI*) to assess the degree of degradation of aviation pumps. To obtain the *DI*, the feature vectors which can characterize the degradation status of the aviation pump should be extracted first. At present, the signal processing technologies, which mainly include the temporal analysis, the frequency analysis and the time-frequency analysis, have been commonly applied in the feature extraction of aviation pump monitoring signals [4]. Although these extracted features can successfully distinguish different failure modes of the aviation pump, the previous studies have shown that the individual time domain, frequency domain or time-frequency domain indicators are difficult to effectively characterize the deterioration degree of the pump [5]. Thus, multi-source feature extraction combining with the above three methods is studied to solve this problem. As indicated in our previous research [5], the multi-domain features, which are acquired from the paving of ensemble empirical mode decomposition (EEMD) based on the discharge pressure signal, can characterize fault severity of the pump accurately. Further, when the dimensionality of these features is reduced by the PCA method, the obtained features are more sensitive for fault severity recognition. As a result, the multi-domain features after reducing dimensionality are considered as feature vectors in this research. After obtaining the feature vectors, it is vital to discover the method of translating these feature vectors into the *DI*. Until now, the main transformation methods include fuzzy c-means (FCM) [6,7,8], self-organization mapping (SOM) [9,10] and Gaussian mixture model (GMM) [11,12]. Among these methods, both normal and final failure data are utilized when using FCM algorithms to calculate the *DI*. Nevertheless, the aviation pump, as a complex mechatronics component, has many different failure modes. This means that the extracted failure features may not be identical. Further, the obtained *DI* will be different, which will greatly affect the evaluation accuracy. Comparing with FCM, the SOM-based methods only need normal data to acquire the *DI*, but the key parameters of SOM network need to be set in advance based upon the experience [12]. Hence, the *DI* originated from SOM is unstable in some cases. Different from SOM, GMM can theoretically estimate any probability distribution function (PDF) to avoid the problem completely [12]. Moreover, only health state data is needed when performing GMM-based degradation assessment. Accordingly, some researchers have applied GMM to calculate the *DI* in the performance degradation evaluation of bearings and hydraulic servo system successfully, but few studies have been done on performance degradation assessment of the aviation pump based on GMM. Besides, there are still some deficiencies among the existing methods for calculating the *DI* based on GMM. For instance, some methods are only suitable for processing single-dimensional features, and some others need to set parameters artificially according to experiences. Therefore, these methods can hardly be applied in the calculation of *DI* when performing the degradation assessment of the pump.

After obtaining the *DI* sequences of an aviation pump, the construction of the prediction model became another challenge in this work. At present, many modeling methods, including autoregressive moving average model (ARMA) [13,14,15], Kalman filtering [16,17], artificial neural network (ANN) [18,19,20], long short-term memory (LSTM) network [21,22] have been successfully applied in degradation prediction of other rotating machines. Although these methods have achieved excellent performance, there still exist some insufficiencies. For instance, ARMA, ANN and LSTM need abundant of training samples, and Kalman filtering depends on accurate mathematical models [12]. However, the aviation pump, as a highly integrated mechatronics component, is hard to characterize by an accurate mathematical model. In addition, the model accuracy may be affected by the weak generalization ability of ANN. Different from the above methods, the support vector regression (SVR) [23], which has the ability of superior generalization in case of small training samples, has been recently used in the field of performance prediction [24,25,26,27]. Though SVR-based methods have shown excellent performance, there are still some problems to be solved. On the one hand, the prediction accuracy is closely related to the number of the observations (inputs) of the SVR model, so the number of the inputs of SVR model needs to be determined reasonably. On the other hand, the selection of the internal parameters of SVR model also affects the prediction accuracy significantly. This means that the optimization of parameters is crucial. Moreover, most of the SVR-based prediction methods use the online data or historical data independently. In fact, both two kinds of data can provide useful information for prediction. To solve problems mentioned above, an optimized SVR model, which considers selection of inputs, optimization of internal parameters and utilization of online and historical data, is constructed to achieve higher prediction accuracy in this work.

The main contributions of this paper are as follows. Firstly, a performance degradation prediction method is proposed for the aviation pump. Secondly, considering that self-information quantity can describe the uncertainty of system by measuring the average information quantity contained in probability distribution, a new *DI*, namely self-information quantity based on GMM, is constructed to track the trend of performance degradation of the pump. To achieve better prediction performance, phase space reconstruction is first adopted to determine the inputs of the SVR model, then a new optimization method, which combines particle swarm optimization (PSO) with grid search (GS), is developed to select the internal parameters of the SVR model. Subsequently, given that the historical data and online data can provide overall trend information and real-time information, respectively, a hybrid SVR model, combining historical data-based SVR and online data-based SVR, is established. Finally, the degradation processes of the pump are reliably estimated via the constructed SVR model. The proposed scheme is validated using the full life cycle experimental data of the aviation pump.

This paper is organized as follows: The proposed performance degradation prediction method is detailed in Section 2. Section 3 verifies the effectiveness of the proposed method using the experimental data. Section 4 gives some comparisons and discussions. Conclusions are drawn in Section 5.

## 2. The Performance Degradation Prediction Method

**Figure 1:**
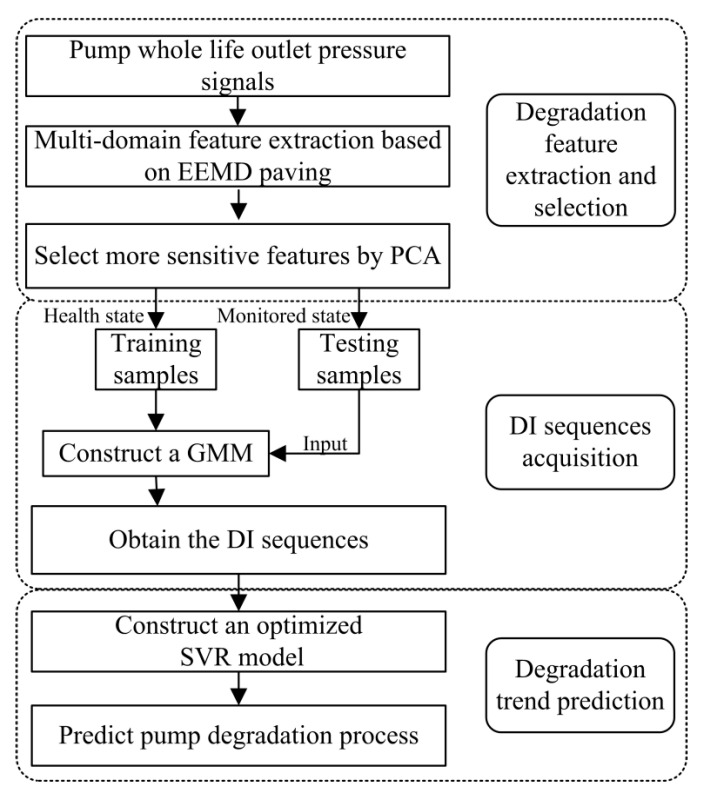
Framework of the proposed performance degradation prediction scheme.

The presented performance degradation prediction scheme consists of the following three steps: multi-domain degradation feature extraction and selection, *DI* sequences acquisition and degradation trend prediction. The details of each step is described in the following subsections. The flowchart of the proposed method is illustrated in Figure 1.

### 2.1. Degradation Feature Extraction and Selection

It follows from previous studies that the failure characteristics can be reflected in pump discharge pressure signals when a pump fails [5,28]. Hence, we can extract useful fault features from pump outlet pressure signals. This section first presents a brief discussion on multi-domain degradation feature extraction via aviation pump pressure signals.

As is well known, the aviation pump is installed near aircraft engines, resulting in violent vibration and severe fluid-solid coupling. This makes pump discharge pressure signals have distinct non-stationary characteristics. Under this condition, the single temporal or frequency analysis is insufficient. Thus, the time-frequency analysis methods are adopted to solve this insufficiency [28,29]. Comparing with other time-frequency methods, EEMD can adaptively decompose according to the local scale of the signal itself and has obvious advantages of processing the non-stationary signals. Therefore, EEMD has been widely used in feature extraction of pump outlet pressure signals.

Much research indicates that these EEMD-based extracted features are enough to diagnose different failure modes effectively, but it will result in low recognition accuracy when utilizing simple time domain or frequency domain or time-frequency domain features to identify different fault severity. In addition, the current methods still have the disadvantage that the intrinsic mode functions (IMFs) information has not been fully explored [9]. For these reasons, this research investigates the EEMD-based multi domain features extraction considering the combination of the above three kinds of features.

As indicated in our previous research [5], the first few IMFs concentrate most of the energy of the signal, so we can choose the appropriate IMFs for signal analysis. To determine the number of proper IMFs, some IMFs are first reconstructed. Secondly, the correlation coefficient between the original signal and the reconstructed signal is computed. Lastly, the proper IMFs can be acquired when the calculation result is bigger than a given threshold, which is usually set to be 0.95 [30]. These selected IMFs are named EEMD paving in this research. Table 1 shows multi-domain features obtained from EEMD paving. Here *T* is the number of selected data points, *x_q_* is a IMF series, *N* is the number of the marginal spectrum lines, *f_z_* and *b_z_* are frequency and amplitude of the *z*th line in a marginal spectrum, *E_k_* represents the *k*-th IMF’s feature energy, *E* is the sum of feature energy of all the selected IMFs.

Previous studies have shown that the sensitivity and stability of commonly used time-domain features are different for identifying failure modes of the pump. For instance, some indicators have obvious advantages of detecting early faults, but the stability of indicators will decrease with the increase of the fault severity. Accordingly, both sensitivity and stability should be considered when selecting indicators. Based on above analysis, seven chosen time-domain features, including square root amplitude value, skewness index, kurtosis index and so on, have been shown in Table 1. Supposing that the number of selected IMFs is *n*, there will be 7*n* time-domain features. In general, time-domain indicators reflect changes of the signal amplitude in the time domain. However, as the degradation degree of pump increases, not only will the time-domain amplitude of the signal change, but frequency and energy distribution of the signal changes as well. Hence, some frequency domain and time-frequency domain indicators listed in Table 1 are introduced to describe this change of distribution.

Though these features extracted from EEMD paving can characterize a pump’s health status from different aspects, it is not the case that all features are equally sensitive to deterioration assessment. Moreover, previous studies [24,26] have proven that too many inputs will significantly increase the computational burden and reduce the evaluation accuracy. Thus, PCA method is employed to capture the most sensitive degradation features, and these optimized features will be used to obtain the *DI* sequences of the pump.

### 2.2. DI Sequences Acquisition Based on GMM

After obtaining the degradation features, the critical task is to transform these features into the reasonable *DI* which can quantitatively describe the degradation degree of the pump. Among the commonly used transformation methods, clustering-based methods needs both normal and failure data. Other methods only require the health data, but some key parameters need to be set artificially. Accordingly, these methods can hardly be applied in the acquisition of the *DI* due to the uncertainty of the ultimate failure of the aviation pump. To solve the above problem, GMM, which considers the distribution characteristic of the features extracted from pump outlet pressure signals, is employed to capture the *DI* in this section.

#### 2.2.1. Brief Description of GMM

Given a *r*-dimensional dataset ***G*** = {***G***_1_, ***G***_2_, …, ***G***_*n*′_}, its PDF can be characterized by a single Gaussian density function *N*(***G***, ***μ***, **∑**) when the dataset has an approximately ellipsoidal distribution in high-dimensional space. However, it is not the case that the data is always ellipsoidal. Under this circumstance, it is difficult to describe the distribution of the data accurately using a single Gaussian density function. Therefore, GMM, which combines several single Gaussian models with different weights, is developed to solve this problem. Based on the above analysis, a GMM can be described as:

(1) $$\begin{array}{l} P(G)=\sum_{h=1}^{M}w_{h}P_{h}(G)=\sum_{h=1}^{M}w_{h}N(G,\mu_{h},\sum_{h})\\ N(G,\mu_{h},\sum_{h})=\frac{1}{\sqrt{{\left(2\pi \right)}^{r}\left|\sum_{h}\right|}}\exp(-\frac{1}{2}{\left(G-\mu_{h}\right)}^{T}\sum_{h}{}^{-1}(G-\mu_{h})) \end{array}$$

where *M* is the number of the mixture model, *w_h_* represents the weight of each Gaussian model and satisfies $\sum_{h=1}^{M}w_{h}=1$, $P_{h}(G)=N(G,\mu_{h},\sum_{h})$ is the PDF of the *h*-th Gaussian model, ***μ**_h_* and $\sum_{h}$ are the mean vector and covariance matrix of the *h*-th Gaussian model, respectively.

From Equation (1), it can be found that the model performance is closely related to the parameters *w_h_*, $\mu_{h}$ and $\sum_{h}$, so the setting of these parameters becomes very important. So far, the maximum likelihood method has been commonly applied in parameters estimation because of its remarkable performance. In general, the optimized parameters are obtained by differentiating the likelihood function in the maximum likelihood method. However, the likelihood function of the parameters of GMM is the logarithm of the sum, it is difficult to get satisfactory results when using traditional direct derivative method. Therefore, the Expectation Maximum (EM) algorithm is used to solve the parameters of GMM in this work.

#### 2.2.2. *DI* Obtained from GMM

As previously noted, GMM can be used to transform the extracted multi-domain degradation features into the *DI* of the aviation pump. In this section, the definition process of the *DI* is described in details.

Firstly, multi-domain degradation features are extracted from pump discharge pressure signals of health state. Then, the sensitive features are selected as training samples for establishing a GMM, and this GMM will be as a benchmark for evaluating the performance of the pump. In a similar way of obtaining the features, assuming that ***G***_*i*′_ is feature vectors obtained from test signals and can be considered as the testing samples, *P*(***G***_*i*′_) will represent the probability that the samples ***G***_*i*′_ generated by GMM constructed by the samples of health state. If the samples are achieved from the signals of degradation state, the value of *P*(***G***_*i*′_) should be less than the output value of health state samples in this GMM. In other words, the value of *P*(***G***_*i*′_) should be smaller than a certain threshold. Thus, *P*(***G***_*i*′_) can characterize the extent to which the tested data deviates from the health condition. Namely, *P*(***G***_*i*′_) can be considered as a *DI*. However, we usually hope that the changes of the *DI* is little in the health state, and the *DI* changes greatly in the degradation state. In addition, a higher *DI* usually represents a failure state, whereas a lower *DI* indicates a normal state. Considering that self-information quantity can describe the uncertainty of system by measuring the average information quantity contained in probability distribution, in a other word, the greater probability, the smaller uncertainty, and thus the smaller self-information quantity. Based on the above analysis, self-information quantity based on GMM is defined as the *DI* in this study below:

(2) $$DI=-\ln(P(G_{i^{\prime }}))$$

**Figure 2:**
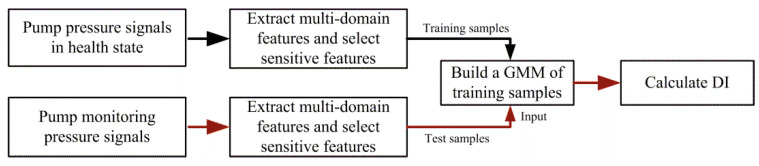
Flowchart of the acquisition process of the *DI*.

The flowchart of the proposed calculation method of the *DI* is presented in Figure 2.

### 2.3. Degradation Prediction Based on Optimized SVR Model

In the previous section, a GMM-based acquisition method of the *DI* has been proposed. In this method, the *DI* which can quantitatively characterize the severity of the deterioration is defined. Generally speaking, with the gradual deterioration of the aviation pump, the value of the *DI* also gradually increases. For the safe operation of the aviation pump, it is essential to make sure the value of the *DI* is less than a certain threshold. Hence, it is vital to predict *DI* in advance. Considering that the non-linearity characteristics of pump outlet pressure signals, a SVR-based prediction method is proposed in this section as the SVR has the strong ability of processing non-linear data.

#### 2.3.1. The Basic Theory of SVR

The core idea of SVR is to map the data *X* into a high-dimensional space through mapping function φ(x) to find a regression line or a regression hyperplane. Given a dataset {(x_i__*_, y_i__*_), i* = 1, 2, …, *B*}, where x_i__*_ represents an input vector, y_i__*_ is the corresponding expected output value, and *B* is the number of samples, the regression function can be expressed as:

(3) f(x)=ω⋅φ(x)+b

where ω denotes the weight vector and *b* represents the offset value. In order to solve ω and *b*, the slack variables $\xi_{i^{*}}^{*}$ and $\xi_{i^{*}}$ are introduced, and the original problem is transformed into an optimization problem of the objective function as follows:

(4) $$\begin{array}{l} \min J=\frac{1}{2}{\left\|\omega \right\|}^{2}+C\sum_{i=1}^{B}(\xi_{i^{*}}^{*}+\xi_{i^{*}})\\ \{\begin{array}{c} y_{i^{*}}-\omega \cdot \varphi (x_{i^{*}})-b\leq \varepsilon +\xi_{i^{*}}\\ \omega \cdot \varphi (x_{i^{*}})+b-y_{i^{*}}\leq \varepsilon +\xi_{i^{*}}^{*}\\ \xi_{i^{*}},\xi_{i^{*}}^{*}\geq 0 \end{array} \end{array}$$

where *C* is a positive constant which penalizes the errors larger than ±*ɛ* using *ɛ*-insensitive loss function.

After obtaining the optimized solution of Equation (4), the regression function can be described as:

(5) $$f(x)=\sum_{i^{*}=1}^{B}(\alpha_{i^{*}}-\alpha_{i^{*}}^{*})(\varphi (x_{i^{*}}),\varphi (x))+b$$

In Equation (5), the kernel function $K(x_{i^{*}},x)=(\varphi (x_{i^{*}}),\varphi (x))$ is employed to compute the inner product in case of non-linear support vector regression. In other words, the non-linear support regression function can be written as:

(6) $$f(x)=\sum_{i^{*}=1}^{B}(\alpha_{i^{*}}-\alpha_{i^{*}}^{*})K(x_{i^{*}},x)+b$$

As shown in Equation (6), the selection of kernel function directly affects the performance of the SVR model. A sea of studies have indicated that satisfactory results can be obtained when Gaussian kernel function is chosen in SVR model [24,25,26,27], so a Gaussian kernel function is adopted in this study.

#### 2.3.2. Optimization of SVR Prediction Model

##### Determination of Inputs of SVR Model

It follows from previous researches that the prediction performance of the SVR model is closely related to the inputs of the model. To achieve the deterioration prediction successfully, it is vital to determine the number of the inputs which are used for predicting the future values. In this work, the key to solving the above problem is the selection of the embedding dimension and delay time of the *DI* sequences. According to the phase space reconstruction theory, if the time series is regarded as being generated by a deterministic nonlinear system, the reconstructed high-dimension vectors can restore the original system when selecting the appropriate embedding dimension and delay time. From this aspect, the problem of the model inputs selection can be equivalent to the solving of the parameters of phase space reconstruction.

The commonly used methods for determining the delay time mainly include the autocorrelation function method [31], the average displacement method [32], the complex autocorrelation function method [33] and the mutual information function method [34]. As the mutual information function method considers both linear and nonlinear factors, the mutual information method is adopted to determine the delay time in this paper. For a more detailed description of mutual information method, the reader is referred to [34]. After choosing a reasonable delay time, the embedding dimension needs to be determined. At present, some methods, such as the geometric invariant method, the false nearest neighbor method and the pseudo nearest neighbor point method (CAO method), have been used to select the embedding dimension. Among these methods, the CAO method is not sensitive to noise and only need the delay time in the calculation process. Therefore, the CAO method [35] is used to determine the embedding dimension in this section. Next, the calculation process of the embedding dimension is given.

For a given time series {$x_{1},x_{2},\ldots ,x_{N^{*}}$}, a sequence of vectors in a new space can be reconstructed as: $y_{i\left(m\right)}=\{x_{i},x_{i+\tau },\ldots x_{i+(m-1)\tau }\},i=1,2,\ldots ,N_{m}$, where *N_m_* is the length of the reconstructed vector series, *m* is the embedding dimension, τ is the delay time.

Firstly, a variable a(i,m) is defined as:

(7) $$a\left(i,m\right)=\frac{{\left\|y_{i}(m+1)-y_{n(i,m)}(m+1)\right\|}_{\infty }}{{\left\|y_{i}(m)-y_{n(i,m)}(m)\right\|}_{\infty }}$$

where ${\left\|•\right\|}_{\infty }$ is the maximum norm, *n*(*i*,*m*) is an integer which make $y_{n(i,m)}(m)$ closest to $y_{i}(m)$ in *m*-dimensional phase space.

Then, based on Equation (7), a new variable is given as:

(8) $$E(m)=\frac{1}{N^{*}-m\tau }\sum_{i=1}^{N^{*}-m\tau }a(i,m)$$

From Equation (8), it can be found that the value of *E*(*m*) is only related to the embedding dimension *m* and the delay time *τ*. In order to study the changing law of *E*(*m*) when the embedding dimension increases from *m* to *m* + 1, the variable *E*_1_(*m*) is defined as follows:

(9) $$E_{1}\left(m\right)=\frac{E(m+1)}{E(m)}$$

In Equation (9), if the embedding dimension *m* is larger than a certain value *m*_0_, the value of *E*_1_(*m*) no longer changes, then (*m*_0_ + 1) will be the minimum embedding dimension. However, it is difficult to accurately determine whether the sequence *E*_1_(*m*) is slowly increasing or has stopped changing [22]. As a result, CAO method has provided additional judgment criteria, namely:

(10) $$\begin{array}{l} E^{*}(m)=\frac{1}{N^{*}-m\tau }\sum_{i=1}^{N^{*}-m\tau }\left|x_{i+m\tau }-x_{n(i,m)+m\tau }\right|\\ E_{2}(m)=\frac{E^{*}(m+1)}{E^{*}(m)} \end{array}$$

As the delay time and embedding dimension are determined, the input number of the SVR prediction model is selected.

##### Internal Parameters Optimization of SVR Model

As previously noted, the SVR model performance is also closely associated with three internal parameters, which are regularization parameter *C*, kernel function parameter *σ* and *ε*-insensitive loss function parameter *ε*, so it is important to properly determine these three parameters. Until now, much research has been done to select the proper parameters. However, some insufficiencies still exist. For instance, some methods are essentially based on the principle of exhaustion, which will make the search process very time-consuming, and some others have no need to traverse all parameter groups but sometimes can easily produce local optimum [36]. Therefore, a new method needs to be developed for parameters optimization in this section.

It follows from previous studies that the model’s accuracy based on direct GS method is low in most of search intervals but the accuracy will be significantly higher in a specific interval. Thus, if we can pre-locate an optimized interval of GS, the search efficiency and the probability of obtaining optimal parameters will increase greatly. To solve this problem, PSO algorithm, which has strong global optimization ability, is first employed to determine three parameters. In this paper, these parameters obtained from PSO method are regarded as first-time optimal parameters. For the purpose of increasing the possibility of capturing the optimal parameters, the searching interval will be relatively enlarged when using PSO method. In addition, to suppress the effects of the randomness, the algorithm operates *h* times repeatedly. In this research, *h* is set to 5 based on reference [5]. After obtaining *h* first-time parameters set of PSO, the final optimal intervals of parameters *C* and *σ* can be defined as follows:

(11) $$\begin{array}{l} \left[C_{\min}^{*},C_{\max}^{*}]=[2^{\left\lfloor \log_{2}(C_{\min})\right\rfloor },2^{\left\lceil \log_{2}(C_{\max})\right\rceil }\right]\\ \left[\sigma_{\min}^{*},\sigma_{\max}^{*}]=[2^{\left\lfloor \log_{2}(\sigma_{\min})\right\rfloor },2^{\left\lceil \log_{2}(\sigma_{\max})\right\rceil }\right] \end{array}$$

where *C*_min_, *C*_max_ and *σ*_min_, *σ*_max_ represent the minimum and maximum of the parameters *C* and *σ* obtained from PSO. ⌊⌋ and ⌈⌉ stand for round down and round up operations to the nearest integer, respectively.

In general, the fluctuation of parameter *ε* is small during the optimization process, so the mean of obtained *ε* based on PSO will be considered as the optimal parameter of *ε* in SVR model, namely:

(12) $$\varepsilon^{*}=\frac{\sum_{k^{\prime }=1}^{h}\varepsilon_{k^{\prime }}}{h}$$

Subsequently, GS method is adopted to select more reasonable values of *C* and *σ* based on the obtained optimal intervals shown in Equation (11). To achieve satisfactory results, the search step of GS method is set as small as possible and K-fold cross-validation scheme is used to evaluate the performance of the model based on the obtained parameter sets. When the mean square error (MSE) of the prediction values is smaller than a given threshold, the algorithm stops. At this time, the obtained parameters will be used to construct the optimized SVR model.

##### Utilization of On-Line Data and Historical Data

In general, the SVR model constructed by online data can capture the short-term deterioration trend, however, the prediction accuracy of online data-based SVR model will decrease greatly when the data changes suddenly. Meanwhile, the SVR model trained by historical data can provide overall trend information of the full life cycle, but it cannot make full use of the real-time information. Consequently, a hybrid SVR model, combining online data-based SVR and historical data-based SVR, is constructed to predict the degradation trend of the pump. Supposing *l*_1_ and *l*_2_ are prediction results of *DI* of the pump based on two kinds of SVR model, respectively, the ultimate prediction result can be captured by weighing the two results as follows:

(13) $$DI_{t}=\alpha_{1t}\cdot l_{1t}+(1-\alpha_{1t})\cdot l_{2t}$$

where *DI_t_* is the ultimate prediction value of *DI* at moment *t*, *l*_1*t*_ represents the prediction value of online data-based SVR at moment *t*, *l*_2*t*_ stands for the prediction value of historical data-based SVR at moment *t*, $\alpha_{1t}$ is the weight of the SVR model constructed by online data with a range of 0–1. In this paper, prior knowledge-based method is adopted to determine the weights of forecasting period. In the process of assigning weights, it mainly depends on the following principles: (1) The weight value should be greater than or equal to 0; (2) The weights of the online model and the historical model are equal when predicting the first value; (3) As the time increases, the weights of the historical model should gradually increase; (4) When time approaches infinity, the weight of the historical model should approach 1. Based on these principles, the weight $\alpha_{1t}$ is given as follows:

(14) $$\begin{array}{cc} \alpha_{1t}=1-\frac{2}{\pi }\arctan(t) & t=1,2,\ldots ,H \end{array}$$

where *H* is the number of prediction steps. It can be easily proved that the defined $\alpha_{1t}$ satisfies the above four conditions.

## 3. Results and Experimental Validation

### 3.1. Experimental Platform

**Figure 3:**
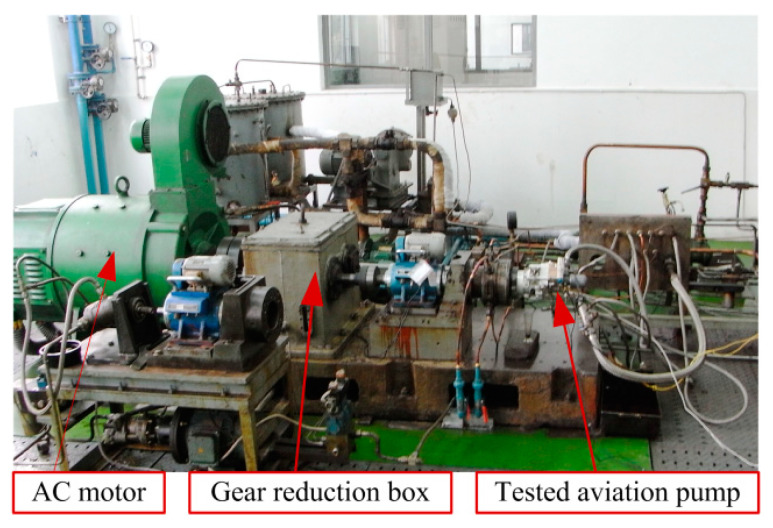
Aviation hydraulic pump test stand.

To verify the effectiveness of the proposed performance degradation prediction method, full life cycle experiments were performed on an aviation pump experimental platform shown in Figure 3. In the experimental platform, an actual aviation pump was driven by a 45 kWAC motor. The rated pressure of the pump was 21 MPa and the rated speed was 4000 r/min. A discharge pressure sensor (0–30 MPa) and a return oil flow sensor were used to collect outlet pressure signals and the return oil flow, respectively. Data acquisition system composed of an industrial computer, a National Instruments (NI, Austin, TX, USA) USB-6221 board, signal conditioning equipments and data collection software developed based on NI LabVIEW^®^ 8.6. The pressure data sampling rate was 2 kHz, and the data was recorded every one hour. Each set of data collection lasted for one minute. When the aviation pump operated 1063 h, the pump was considered as a total failure in case of the monitoring return oil flow exceeding the failure threshold 2.8 L/min. After disassembling the tested pump, it can be found that the clearance between plunger ball head and slipper socket exceeded a given threshold 0.2 mm. At this time, the experiments stopped. A total of 1063 data sets were collected for the entire experiment. In each data set, the signal data is divided into two segments, one part is used as the online data, and the other part is treated as the historical data.

### 3.2. Experimental Results and Analysis

**Figure 4:**
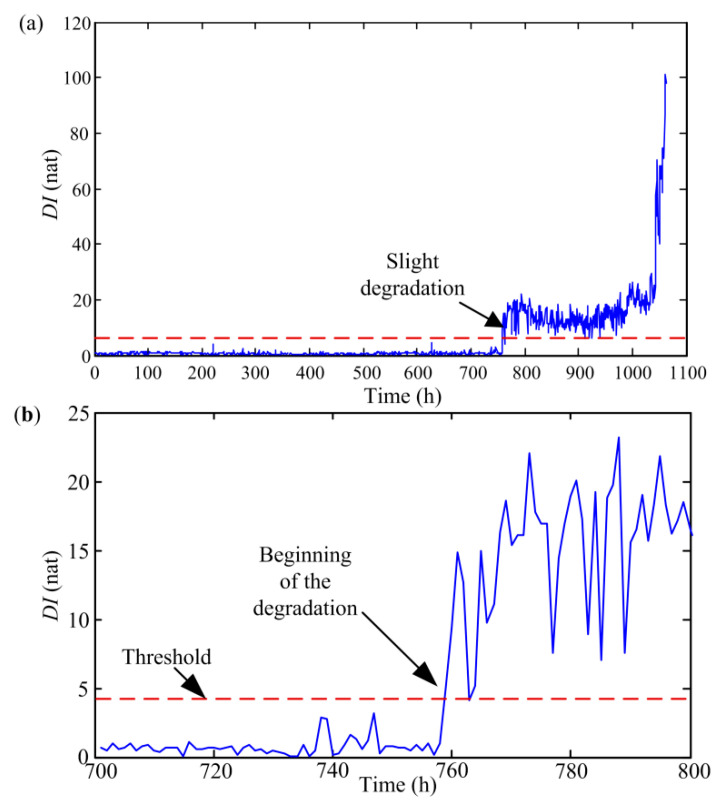
*DI* sequences of the aviation pump based on GMM: (**a**) full life cycle and (**b**) partial enlargement drawing.

In this section, the historical data is first analyzed to illustrate the calculation process of the *DI* of the pump. Firstly, based on the proposed feature extraction method, multi-domain features are extracted from 1063 data sets and the number of the obtained features is 32 in each data set. To select more sensitive features and decrease the computational burden, PCA is used to reduce the dimension of features. In general, the threshold of PCA method is set to 0.85 [37]. Through calculation, we find that the cumulative contribution rate has exceeded 95% when the first seven principal components is chosen. Hence, these seven principal components are used to replace the original 32 multi-domain features of each data set and regarded as a sample to calculate the *DI*. On the basis of this, a total of 1063 samples are obtained. Among these samples, the first 200 samples, obtained from health state signals, are selected as training samples to construct a GMM. According to the experience, three to five Gaussian functions are enough when approximating the PDF of the extracted features. Thus, the number of Gaussian models participating in combination is set to 4 in this paper [12], and the weights of four Gaussian models through EM calculation are 0.1219, 0.5166, 0.2081, and 0.1534, respectively. After determining parameters of GMM, all samples are input to the constructed GMM, and the *DI* is computed based on Equation (2). Figure 4 shows the *DI* curves of all samples.

From Figure 4, it can be seen that the curve remains stable for a long time firstly and suddenly changes at the 759th point. From point 759, the value of *DI* increases significantly, which indicates that the aviation pump has entered an early stage of degradation. To verify the accuracy of diagnosing an early failure of the pump based on *DI*, the data sets 758 and 759 are analyzed.

**Figure 5:**
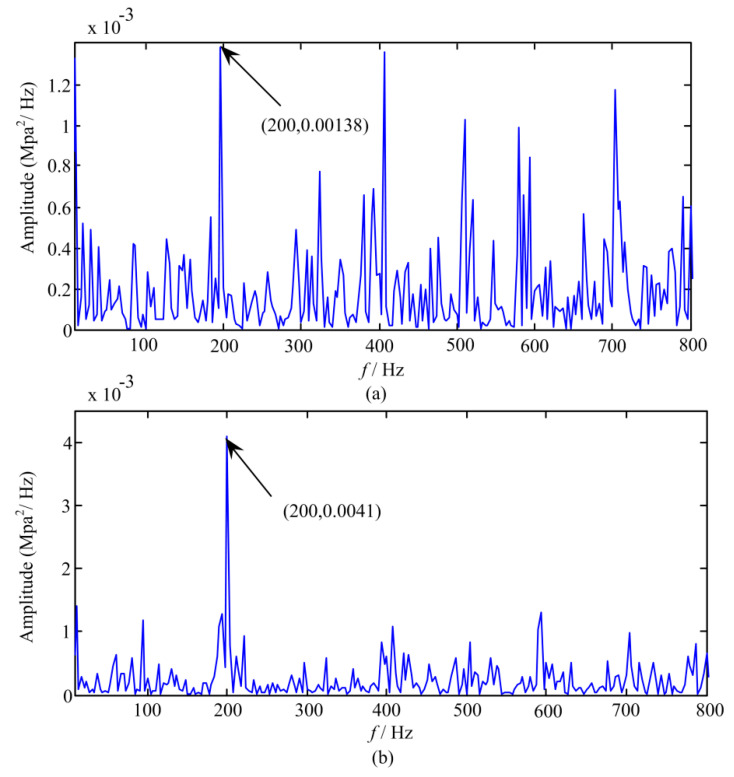
Power spectra of pressures signals obtained from (**a**) data set 758 and (**b**) data set 759.

Figure 5 depicts the power spectra of pressure signals obtained from two data sets. As illustrated in Figure 5, the amplitude of 200 Hz in dataset 759 increases by about three times and the amplitude of 0–200 Hz also increases significantly compared with the results in dataset 758. According to the previous failure mechanism analysis of the pump, it can be known that the amplitudes at fundamental frequency (66.7 Hz) and its multiples will rise with the increase of the fault severity. Moreover, comparing with the results in data set 759, it is also found that the amplitudes at fundamental frequency and third harmonic are also obviously lower in the data before dataset 758. Consequently, it can be inferred that the pump has undergone an early deterioration from point 759. Meanwhile, it also proves that the proposed *DI* namely self-information quantity based on GMM can accurately identify the early degradation of the pump. Furthermore, as indicated in Figure 5, the values of self-information quantity obtained from the data sets after entering the early degraded state are greatly larger than those in health state, which shows that we can set up an appropriate threshold to distinguish between health and early degeneration state.

After the pump enters an early degeneration stage, the changes of the *DI* are relatively stable from points 759 to 990. Subsequently, the *DI* values obviously increase again from the 990th point, which shows that the degradation severity of the pump is increasing. Further, the values of *DI* rise rapidly from point 1043. Currently, it can be indicated that the pump has entered a critical failure stage.

**Figure 6:**
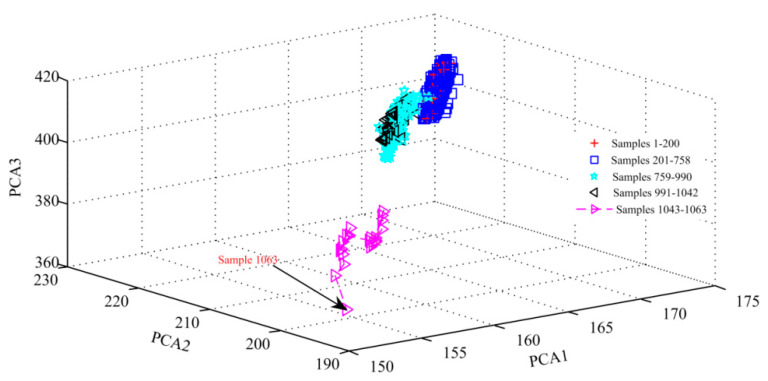
Spatial distribution of all samples when using the first three principal components.

Figure 6 displays the spatial distribution of all samples graphically when using the first three principal components. As can be seen from Figure 6, the distribution of samples 201–758 basically coincides with that of training samples since samples 201–758 are also obtained from pressure signals in a healthy state. The distances between the sample point and training sample sets are increasing gradually as the pump goes from a healthy state to an early degradation state, severe degradation and eventually failure. This is basically consistent with the change of the *DI*, which once again proves that the proposed *DI* based on GMM can effectively characterize the degradation degree of the aviation pump.

**Figure 7:**
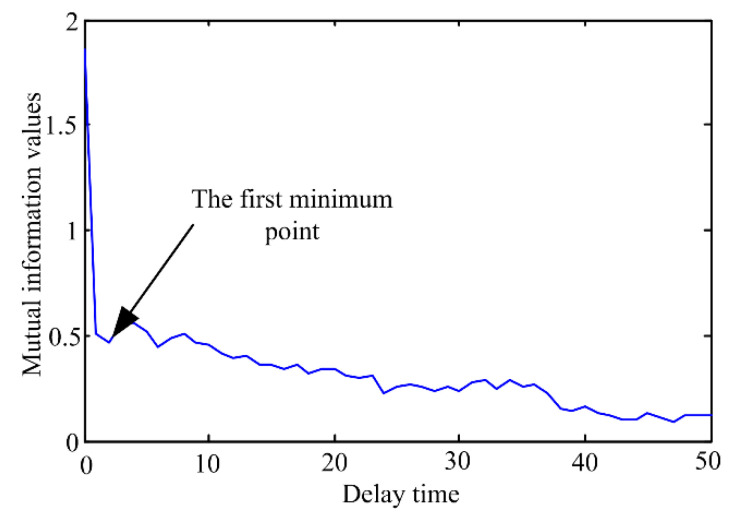
Selection of the delay time by the average mutual information method.

After obtaining the *DI* time series of the pump, optimized SVR model is constructed to achieve multi-step ahead prediction. From Figure 4, we can find that the pump is in healthy state before point 759 for historical data. Meanwhile, similar conclusions can be obtained by analyzing the online data, so this paper mainly focus on points 759–1063. As described in Section 2.3, the inputs of the SVR model needs to be determined firstly. To solve this problem, the average mutual information method and CAO method are separately adopted. Figure 7 illustrates the selection results of delay time based on the average mutual information.

**Figure 8:**
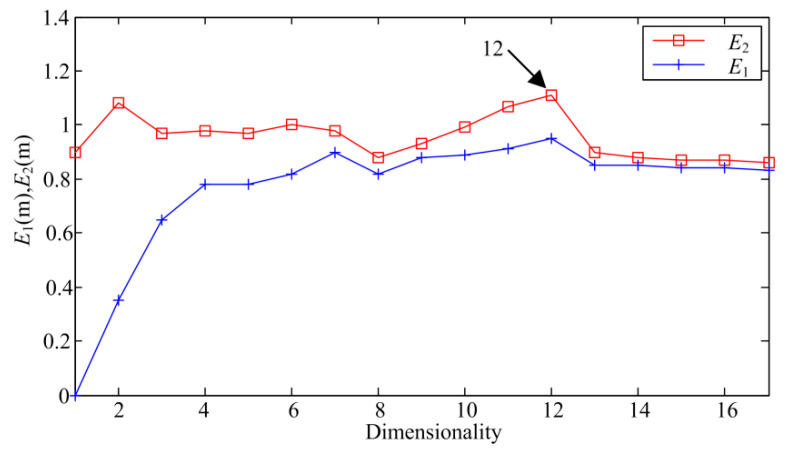
Selection of the embedding dimension by CAO method.

As depicted in Figure 7, the first minimum point of the curve appears at τ=2, so the delay time is set to 2. After the delay time is determined, the CAO method is utilized to capture the embedding dimension *m*. Figure 8 depicts the change curve of variables *E*_1_(*m*) and *E*_2_(*m*) with the increase of the embedding dimension. From Figure 8, it can be observed that the values of *E*_1_(*m*) and *E*_2_(*m*) no longer increase when the embedding dimension *m* = 12. As a result, the embedding dimension *m* is set to 12.

Based upon the obtained parameters τ and *m*, the inputs of the prediction model is determined. Further, the *DI* sequences {*x*_759_, *x*_760_, …, *x*_1063_}, extracted from the historical data, can be reconstructed as follows:

(15) $$\begin{array}{cc} X_{train}=\left[\begin{array}{cccc} x_{759} & x_{761} & \ldots & x_{781}\\ x_{760} & x_{762} & \ldots & x_{782}\\ \vdots & \vdots & \vdots & \vdots \\ x_{1040} & x_{1042} & \ldots & x_{1062} \end{array}\right] & Y_{train}=\left[\begin{array}{c} x_{782}\\ x_{783}\\ \vdots \\ x_{1063} \end{array}\right] \end{array}$$

where *X_train_* and *Y_train_* are the input and target output of the historical data-based SVR model, respectively. The SVR model is then trained by the training samples {*X_train_*, *Y_train_*}. Next, three key parameters *C*, *σ*, and *ε* are optimized to achieve the better performance of the SVR model. According to the proposed optimization method, PSO algorithm is first adopted to obtain the optimized searching intervals. Table 2 presents some parameters of PSO method.

Based on Equations (11) and (12), the optimization intervals of *C* and *σ*, namely *C* ∈ [2^0^,2^6^] and *σ* ∈ [2^−2^,2^2^], are obtained, and ε* = 0.001 will be as the final optimization parameter of *ε*. Compared with the results in Table 2, it can be found that the searching intervals have been reduced greatly. Based upon the obtained optimization intervals, GS method is adopted to select the reasonable values of the parameters *C* and *σ*. In GS method, the search step of *C* and *σ* are small enough, which are 0.1 and 0.01, respectively. In addition, 5-fold cross validation is used to evaluate the performance of the selected parameter sets. Subsequently, the optimal parameters *C* = 22.1, *σ* = 2 are obtained. With the determination of the optimal parameters, the optimization SVR model trained by the historical data is constructed to predict the *DI* values.

**Figure 9:**
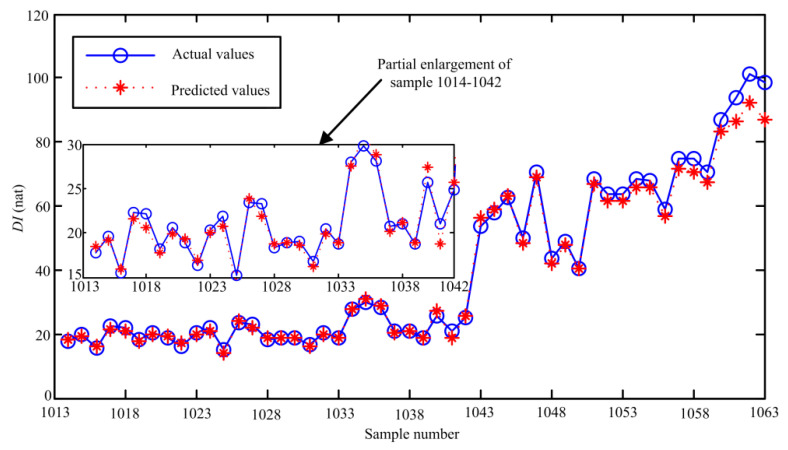
The actual values and predicted results based on the proposed method.

As the prediction steps is set to 50 in this paper, the *DI* time series {x*_759_, x*_760_, …, x*_1013_}, obtained from the online data, is used to train the online data-based SVR model. In a similar way, the original time series can be reconstructed as follows:

(16) $$\begin{array}{cc} X^{*}{}_{train}=\left[\begin{array}{cccc} x_{759}^{*} & x_{761}^{*} & \ldots & x_{781}^{*}\\ x_{760}^{*} & x_{762}^{*} & \ldots & x_{782}^{*}\\ \vdots & \vdots & \vdots & \vdots \\ x_{990}^{*} & x_{992}^{*} & \ldots & x_{1012}^{*} \end{array}\right] & Y^{*}{}_{train}=\left[\begin{array}{c} x_{782}^{*}\\ x_{783}^{*}\\ \vdots \\ x_{1013}^{*} \end{array}\right] \end{array}$$

where X*_train_ and Y*_train_ are the input and target output of the online data-based SVR model, respectively. Based on the proposed optimization method, *C* = 5.1, *σ* = 1 and ε*= 0.001 are selected as the optimal parameters. Next, the first testing sample {[x*_991_x*_993_ … x*_1013_], x*_1014_} is input to the online data-based SVR model to get the predicted value ${\hat{x}}_{1014}^{*}$, then ${\hat{x}}_{1014}^{*}$ is added to the second testing samples to predict the second value ${\hat{x}}_{1015}^{*}$, and so on. Fifty prediction values will be acquired based on the SVR model constructed by the online data. Similarly, 50 prediction values can be obtained when the testing samples are input to the historical data-based SVR model. Subsequently, the final predicted values can be obtained based on Equation (13). The actual values and predicted values of the proposed method are presented in Figure 9.

From Figure 9, it can be found that the change trend of the *DI* curve can be predicted by the optimized SVR model effectively. Meanwhile, we can find that the deviation of the predicted values and actual values of samples 1014–1042 is smaller than that of samples 1043–1063. The possible reason is that samples 1014–1042 are in the same deterioration stage. To quantitatively assess the prediction accuracy, some statistical indexes, including maximum relative error (MRE), average relative error (ARE) and root mean square error (RMSE), are given as:

(17) $$\begin{array}{l} \mathrm{MRE}=\max_{k^{*}\in [1,H]}(\left|f{\left(k^{*}\right)}_{predictd}-f{\left(k^{*}\right)}_{actual}\right|/f{\left(k^{*}\right)}_{actual})\\ \mathrm{ARE}=\frac{1}{H}\sum_{k^{*}=1}^{H}\left(\left|f{\left(k^{*}\right)}_{predictd}-f{\left(k^{*}\right)}_{actual}\right|/f{\left(k^{*}\right)}_{actual}\right)\\ \mathrm{RMSE}={\left(\frac{1}{H}\sum_{k^{*}=1}^{H}{\left(\left|f{\left(k^{*}\right)}_{predictd}-f{\left(k^{*}\right)}_{actual}\right|\right)}^{2}\right)}^{1/2} \end{array}$$

where *H* is the number of the ahead prediction steps, *f*(k*)*_predicted_* is the k*-th predicted *DI* value, and *f*(k*)*_actual_* is the k*-th actual *DI* value when performing *H*-step ahead prediction.

Table 3 presents the calculated statistical indexes of the optimized SVR model. From Table 3, it can be observed that most of errors of 29-step ahead prediction are smaller than that of 50-step ahead prediction. This shows that the prediction error will gradually rise as the prediction steps increase. Nevertheless, the RMSE of 50-step ahead prediction results is only 2.82, it can be concluded that the proposed method can accurately track the change of the degradation status of the aviation pump.

## 4. Comparisons and Discussion

The experimental results have demonstrated that the proposed approach can assess the pump performance degradation effectively and predict the change trend of the degradation status with high accuracy. To further prove its superiority, we make some comparisons in this section. On the one hand, the time domain-based method, FCM-based method, and so on, are compared to verify the advantages of the proposed *DI* when performing degradation assessment. On the other hand, some published methods, including back propagation (BP), GS-based SVR, genetic algorithm (GA)-based SVR, LSTM, among others, are used to compare the performance of predicting the degradation process.

**Figure 10:**
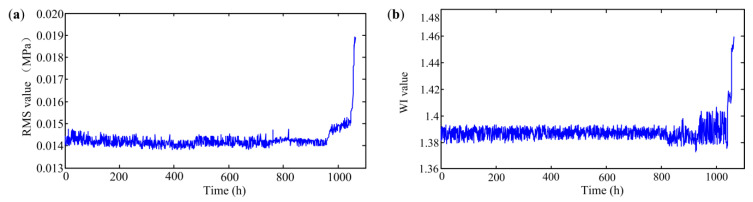
The obtained *DI* based on different time-domain statistical indicators; (**a**) RMS (**b**) WI.

Some commonly used time-domain statistical indicators, such as the root mean square (RMS) value and waveform index (WI), have been widely applied in the performance degradation evaluation of other rotating machinery. Among these methods, it can be found that the RMS value is generally sensitive to wear-related faults and the WI has better stability. To show the effectiveness of the proposed method, these two indexes are first compared with the presented *DI*. Figure 10a and Figure 10b depict the RMS and WI values obtained from the pump’s full life cycle data, respectively.

As illustrated in Figure 10a, the RMS values of the first 980 points fluctuate slightly and the values increase significantly from point 1000. This shows that the RMS-based method is much later than the proposed method in detecting early degradation of the pump. Comparing with Figure 10a, we can find that the WI values of the first 810 samples also fluctuate slightly in Figure 10b. However, the values first decrease and then rise from point 810. This will make it difficult to use the WI to track the development of the degradation degree.

**Figure 11:**
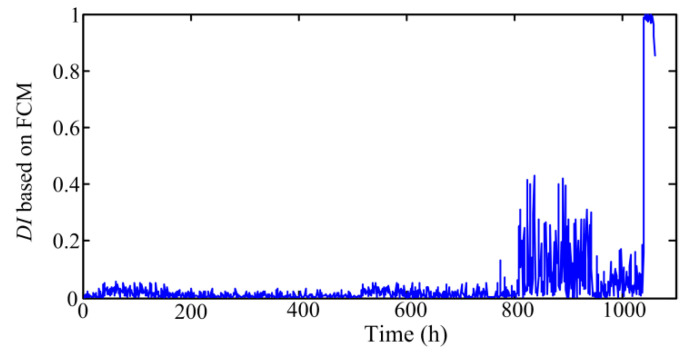
The *DI* obtained from FCM method.

As described in previous section, FCM-based method is also widely employed in performance degradation evaluation of rotating machinery, so it is used to compare with the proposed method in this paper. Figure 11 shows the obtained *DI* based on the FCM method.

From Figure 11, it can be observed that the fluctuation of the *DI* values obtained from FCM is small from sample 1 to sample 810, which means the pump is in healthy state at this stage. From sample 811 to sample 945, the fluctuation of the *DI* values increases greatly. Subsequently, the values begin to decrease from sample 946, and then the values rise sharply from sample 1041, which indicates that the pump begin to enter the near failure stage from point 1041. Comparing Figure 11 with Figure 4, we can find that the pump degradation is also roughly divided into four stages in Figure 11. However, the FCM-based method is later than the proposed method in recognizing the early degradation of pump. Besides, as the deterioration degree increases, the *DI* values obtained from FCM increases first, then decreases, and then increases again. Under this circumstance, it is difficult to track the development of the pump degradation degree by use of the FCM-based *DI*.

**Figure 12:**
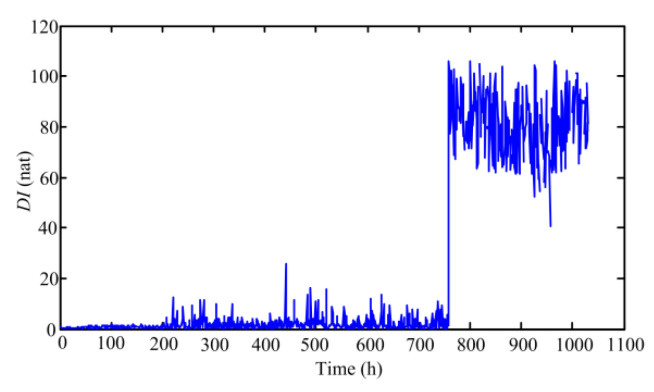
The obtained *DI* using original 32-dimension features.

Next, original 32-dimensionality features are utilized to explore the impact of the feature dimensionality on degradation assessment. Figure 12 shows the obtained *DI* without reducing feature dimensions. From Figure 12, we can find that the *DI* also change greatly at the 759th point. This means that the health state and early degradation can be distinguished. However, we cannot find obvious degradation trend from the curve after point 759. This shows that the *DI* obtained from original 32-dimension features cannot track the degradation state accurately.

**Figure 13:**
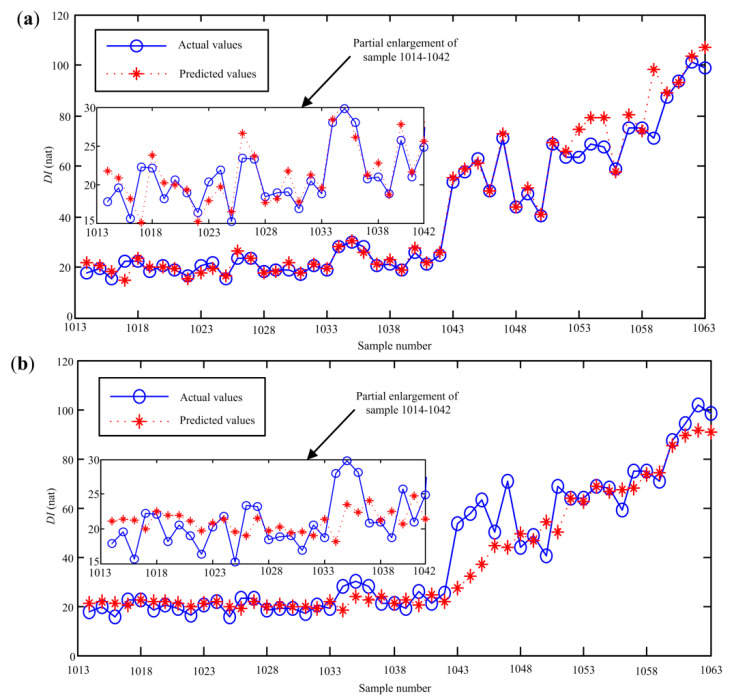

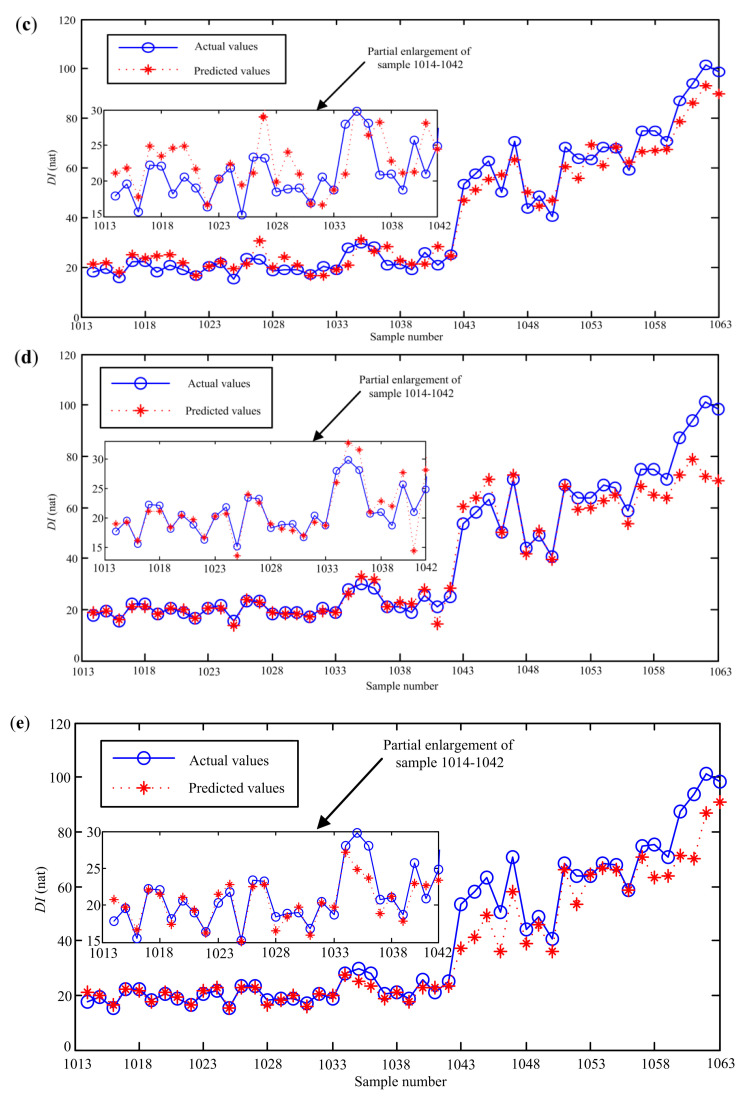
The actual values and predicted results based on different methods; (**a**) BP (**b**) GS-SVR (**c**) GA-SVR (**d**) PSO-SVR (**e**) LSTM.

After achieving the comparisons of the *DI* acquisition methods, some prediction methods are compared with the proposed SVR model. The parameters setting of the compared methods can be found in [5,26]. To avoid the occasionality of single operation, each algorithm is repeated 10 times, and the results with the smallest error is selected for comparison. Figure 13 shows the actual values and the predicted results based on these compared methods. From Figure 13, it can be found that the predicted results based on GS-SVR and GA-SVR, fluctuate more violently than those obtained from the remaining methods when performing 29-step ahead prediction.

Among the remaining three methods, LSTM has the smallest deviations between the actual values and predicted values. This is because samples 1014–1042 are in the same degradation stage, and LSTM can learn the inherent laws of the data better due to the introduction of the gate. As the ahead prediction steps increase, the prediction errors of samples 1043–1063 based on LSTM increase significantly. The possible reason is that the number of the training samples is small and the *DI* sequences of the pump have no obvious periodicity. Meanwhile, we can see that GS-SVR works the worst when performing 50-step prediction, this is because the searching efficiency of direct GS is lower when selecting the optimal parameters of the SVR model. Comparing Figure 13 and Figure 9, it can be seen that the fluctuations of the prediction results shown in Figure 13 are clearly bigger than those obtained from the proposed method. It indicates that the generalization ability of the constructed SVR model is better and thus higher prediction accuracy can be achieved.

To quantify the prediction accuracy of these methods, the statistical indexes of different methods are presented in Table 4. Comparing Table 4 with Table 3, it can be observed that the proposed method works the best, this is because the SVR model has better ability of processing small sample in comparison with BP and LSTM, and the combination of PSO and GS greatly increase the probability of obtaining the optimal parameters. So through comparisons we can get that the proposed SVR model can track the general trend of the performance degradation of the aviation pump better.

## 5. Conclusions

This study proposes a new effective approach for evaluating and predicting the degradation process of the aviation pump. Unlike the traditional failure modes identification and fault severity recognition, this study mainly focuses on the discovery of the methods which can reliably track the degradation status of the aviation pump. Based on the aforementioned illustration, the presented scheme includes an EEMD paving-based multi-domain features extraction, a GMM for performance degradation assessment, and a degradation trend prediction using optimized SVR. According to the experimental results and the comparisons, the following can be concluded:

(1) The multi-domain features extracted from EEMD paving based on pump outlet pressure signals can successfully characterize the degradation degree of the pump than traditional features, such as RMS and WI.
(2) The *DI* derived from GMM can effectively identify and track the current deterioration stage, which enables the determination of the critical fault occurrence accurately and the realization of condition-based maintenance.
(3) The proposed method provides a useful tool for multi-step ahead prediction of the *DI* and has higher accuracy compared to some previously published methods, including BP, GA-SVR, and so on.
(4) As full life cycle experiment of the aviation pump is expensive and very time-consuming, there is only few life samples, which will affect the further verification of the method. Meanwhile, the weights of the models are given according to the experience. In the future, some research will be explored on how to determine the weights more reasonably.

## Figures and Tables

**Table 1:** Multi-domain features extracted from EEMD paving.

Square root amplitude value: ${\left(\frac{1}{T}\sum_{q=1}^{T}\sqrt{\left|x_{q}\right|}\right)}^{2}$	Impulsive index: $\max(\left|x_{q}\right|)/(\frac{1}{T}\sum_{q=1}^{T}\left|x_{q}\right|)$
Shape index: $\sqrt{\frac{1}{T}\sum_{q=1}^{T}x_{q}^{2}}/(\frac{1}{T}\sum_{q=1}^{T}\left|x_{q}\right|)$	Clearance index: $\max(\left|x_{q}\right|)/{\left(\frac{1}{T}\sum_{q=1}^{T}\sqrt{\left|x_{q}\right|}\right)}^{2}$
Crest index: $\max(\left|x_{q}\right|)/\sqrt{\frac{1}{T}\sum_{q=1}^{T}x_{q}^{2}}$	Root mean square frequency: $\sqrt{\sum_{z=1}^{N}f_{z}^{2}b_{z}/\sum_{z=1}^{N}b_{z}}$
Skewness index: $\sum_{q=1}^{T}x_{q}^{3}/T{\left(\sqrt{\frac{1}{T}\sum_{q=1}^{T}x_{q}^{2}}\right)}^{3}$	Centroid frequency: $\sum_{z=1}^{N}f_{z}b_{z}/\sum_{z=1}^{N}b_{z}$
Kurtosis index: $\sum_{q=1}^{T}x_{q}^{4}/T{\left(\sqrt{\frac{1}{T}\sum_{q=1}^{T}x_{q}^{2}}\right)}^{4}$	Frequency variation: $\sum_{z=1}^{N}f_{z}^{2}b_{z}/\sqrt{\sum_{z=1}^{N}b_{z}\sum_{z=1}^{N}f_{z}^{4}b_{z}}$
Hilbert marginal spectrum-based energy entropy: $\begin{array}{cc} -\sum_{k}^{n}p_{k}\mathrm{lg}(p_{k}) & p_{k}=E_{k}/E,E=\sum_{k=1}^{n}E_{k} \end{array}$	

**Table 2:** Parameter settings of PSO method.

Parameter	The Value
The maximum number of generations	200
The number of the particles	20
Learn factors	1.5,1.7
Inertia weight	1
The initial range of *C*	[0,1000]
The initial range of *σ*	[0,100]
The initial range of *ε*	[0.001,1]

**Table 3:** The statistical indexes based onoptimized SVR model.

*H*	MRE	ARE	RMSE
29	0.1079	0.0323	0.85
50	0.1188	0.0363	2.82

**Table 4:** The prediction error comparisons based on different methods.

Methods	*H*	MRE	ARE	RMSE
GS-SVR	29	0.3605	0.1422	3.66
	50	0.4889	0.1464	9.02
GA-SVR	29	0.3605	0.1434	3.74
	50	0.3605	0.1256	5.29
PSO-SVR	29	0.3110	0.0615	1.92
	50	0.3110	0.0779	7.32
BP	29	0.3250	0.078	2.13
	50	0.3887	0.0719	5.27
LSTM	29	0.1686	0.0537	1.68
	50	0.3087	0.0874	7.28
